# Lambda-cyhalothrin and male reproductive dysfunction: neurotoxicity, hypogonadism, erectile dysfunction, and infertility

**DOI:** 10.3389/fpubh.2026.1956109

**Published:** 2026-10-02

**Authors:** Ana-Maria Cretu, Ahmed Adel Mansour Kamar, Alin Ciobica, Dragos Puia, Mara Doroftei, Bogdan Doroftei

**Affiliations:** 1 Origyn Fertility Center, Iași, Romania; 2 Faculty of Medicine, Grigore T. Popa University of Medicine and Pharmacy Iasi, Iași, Romania; 3 Department Biology, Faculty of Biology, Doctoral School, Alexandru Ioan Cuza University, Iași, Romania; 4 Department Biology, Faculty of Biology, Alexandru Ioan Cuza University (UAIC), Iași, Romania; 5 Department of Urology, “Dr. C.I. Parhon” Clinical Hospital, Iași, Romania; 6 Spitalul Clinic de Obstetrică și Ginecologie “Cuza Vodă”, Iași, Romania

**Keywords:** endocrine disruption, erectile dysfunction, lambda-cyhalothrin, male hypogonadism, male infertility, neurotoxicity

## Abstract

Lambda-cyhalothrin is one of the most widely used type II pyrethroid insecticides in agriculture, public health programs, and domestic pest control. Although their acute toxicity in humans is generally considered low, concerns have been raised regarding their potential effects on male reproductive function. Male infertility affects a substantial proportion of couples worldwide, and environmental exposures have been proposed as contributing factors. This review critically examines the available evidence linking lambda-cyhalothrin exposure to male reproductive dysfunction. Experimental studies have reported adverse effects on neurotoxicity, reproductive hormone regulation, testicular function, spermatogenesis, and sperm quality following lambda-cyhalothrin exposure. Human studies have identified associations between urinary pyrethroid metabolites and reduced semen quality, altered reproductive hormones, and sperm DNA damage. However, the available epidemiological evidence remains limited and heterogeneous. Current findings indicate that lambda-cyhalothrin may adversely affect male reproductive function through neurotoxicity, endocrine disruption, oxidative stress, impaired testicular function, and sperm damage. While the biological plausibility of these effects is supported by experimental data, the extent of their contribution to infertility in exposed human populations remains uncertain. Unlike previous reviews, this review proposes an integrated neuroendocrine framework linking central, autonomic, and peripheral neurotoxicity with hypothalamic–pituitary–gonadal axis dysfunction, hypogonadism, erectile dysfunction, and male infertility following lambda-cyhalothrin exposure. This integrated perspective highlights important evidence gaps and may guide future experimental, occupational, epidemiological, and clinical research.

## 1 Introduction

Lambda-cyhalothrin is a synthetic type II pyrethroid insecticide characterized by the presence of an α-cyano group, a structural feature associated with greater neurotoxic potency than type I pyrethroids. Owing to its high insecticidal efficacy, broad-spectrum activity, and relatively favorable acute mammalian safety profile, lambda-cyhalothrin is extensively used in agriculture, vector-control programs, veterinary applications, and residential pest management worldwide. Its widespread use has resulted in frequent occupational, environmental, and domestic exposure (1–3).

From a physicochemical perspective, lambda-cyhalothrin is highly lipophilic, exhibits low water solubility, and strongly adsorbs to soils and organic matter, characteristics that contribute to environmental persistence under certain conditions while limiting mobility in groundwater (3). Human exposure occurs primarily through dermal absorption, inhalation of aerosols or contaminated dust, and dietary ingestion of pesticide residues, with occupational exposure representing the highest-risk scenario because of repeated handling, mixing, spraying, and equipment maintenance (1, 2, 4).

Following absorption, lambda-cyhalothrin undergoes extensive hepatic biotransformation mediated by ester hydrolysis and cytochrome P450 enzymes, producing metabolites that are subsequently conjugated and eliminated mainly through urine and feces (1, 3, 4). Although the parent compound is not considered highly bioaccumulative in humans, repeated or chronic exposure may result in sustained biological effects through recurrent oxidative stress, neurotoxicity, endocrine disruption, and cumulative tissue injury (5–12). These toxicokinetic and physicochemical characteristics provide an important biological context for understanding the potential mechanisms underlying lambda-cyhalothrin-associated male reproductive dysfunction.

Male infertility is a major public health concern. Infertility affects approximately 8–15% of couples worldwide, with male factors contributing to 30–50% of cases and being involved in nearly half of infertile couples (13). Although genetic abnormalities, endocrine disorders, anatomical defects, infections, and lifestyle factors are established causes of impaired fertility, increasing attention has focused on environmental exposures as potentially modifiable contributors to male reproductive dysfunction.

Despite its favorable acute safety profile, concerns have been raised regarding the potential reproductive effects of long-term exposure. Experimental studies have shown that lambda-cyhalothrin may interfere with endocrine function, alter hormone receptor activity, induce oxidative stress, and impair cellular homeostasis (6–12, 14–16). These biological effects are particularly relevant to the male reproductive system, where hormonal regulation and oxidative balance play essential roles in spermatogenesis and fertility.

Evidence from experimental and human studies has also raised concerns regarding adverse reproductive outcomes following lambda-cyhalothrin exposure (6, 7, 14–17). Experimental studies have demonstrated neurotoxicity, endocrine disruption, impaired Leydig cell development, and reduced reproductive competence, while human studies have reported associations between pyrethroid metabolites and altered reproductive hormone concentrations (6, 7, 14–18). In addition, environmental exposure to pyrethroids has been linked to increased sperm DNA damage, a recognized factor associated with reduced male fertility and adverse reproductive outcomes (19). Experimental evidence further supports the potential involvement of lambda-cyhalothrin in sperm and reproductive toxicity (7, 17). Biomonitoring studies have consistently demonstrated measurable pyrethroid exposure in both occupationally exposed individuals and the general population (1, 2, 4).

Although the widespread use of lambda-cyhalothrin has coincided with growing concern regarding male reproductive health, a causal relationship between its exposure and male infertility has not been established. Nevertheless, findings from experimental studies and human investigations have raised concerns regarding the potential effects of lambda-cyhalothrin on spermatogenesis, reproductive hormones, sperm quality, and fertility outcomes.

Accordingly, this review critically examines the available evidence on lambda-cyhalothrin-induced male reproductive dysfunction, with particular emphasis on neurotoxicity, endocrine disruption, hypogonadism, erectile dysfunction, and infertility.

Previous reviews have primarily summarized oxidative stress, endocrine disruption, and semen quality following pyrethroid exposure (6, 15). However, the potential interaction between central, autonomic, and peripheral neurotoxicity, hypothalamic–pituitary–gonadal axis dysfunction, hypogonadism, erectile dysfunction, and male infertility has not been critically synthesized for lambda-cyhalothrin. This review addresses this knowledge gap by proposing an integrated neuroendocrine framework linking these interconnected mechanisms.

### 1.1 Research question

To what extent is lambda-cyhalothrin exposure associated with male reproductive dysfunction, and what evidence supports the mechanistic links between neurotoxicity, hypogonadism, erectile dysfunction, and infertility?

## 2 Methodology and literature search strategy

This narrative review examines the available evidence on lambda-cyhalothrin exposure and male reproductive dysfunction. Relevant articles were identified through searches of PubMed/MEDLINE, Scopus, and Web of Science using combinations of the terms “lambda-cyhalothrin,” “male hypogonadism,” “erectile dysfunction,” “male infertility,” “neurotoxicity,” “endocrine disruption,” “spermatogenesis,” “sperm DNA damage,” “testosterone,” and “occupational exposure.”

The search strategy combined free-text keywords and Medical Subject Headings (MeSH), where applicable, using the Boolean operators AND and OR to optimize retrieval of relevant studies.

Peer-reviewed articles published in English up to July 2026 were considered. Preference was given to human studies evaluating reproductive outcomes, supported by experimental investigations examining testicular function, hormonal changes, and sperm-related effects. Additional references were identified from the bibliographies of relevant articles. Additional relevant studies were also identified through a year-by-year search of Google Scholar. A total of 118 records were identified through database and supplementary searches. After removal of 22 duplicates, 96 records were screened for relevance. Following title and abstract screening, 25 records were excluded, and 71 full-text articles were assessed for eligibility. Of these, 10 articles were excluded because they did not meet the eligibility criteria, and 61 publications were included in the final narrative synthesis. The literature search and study-selection process is summarized in Figure 1. Conference abstracts, duplicate publications, non-English articles, and studies not directly relevant to the objectives of this review were excluded. Literature screening and study selection were performed independently by two authors (A. A. M. K. and M. D.), with any discrepancies in study inclusion resolved through discussion until consensus was reached.

**Figure 1:**
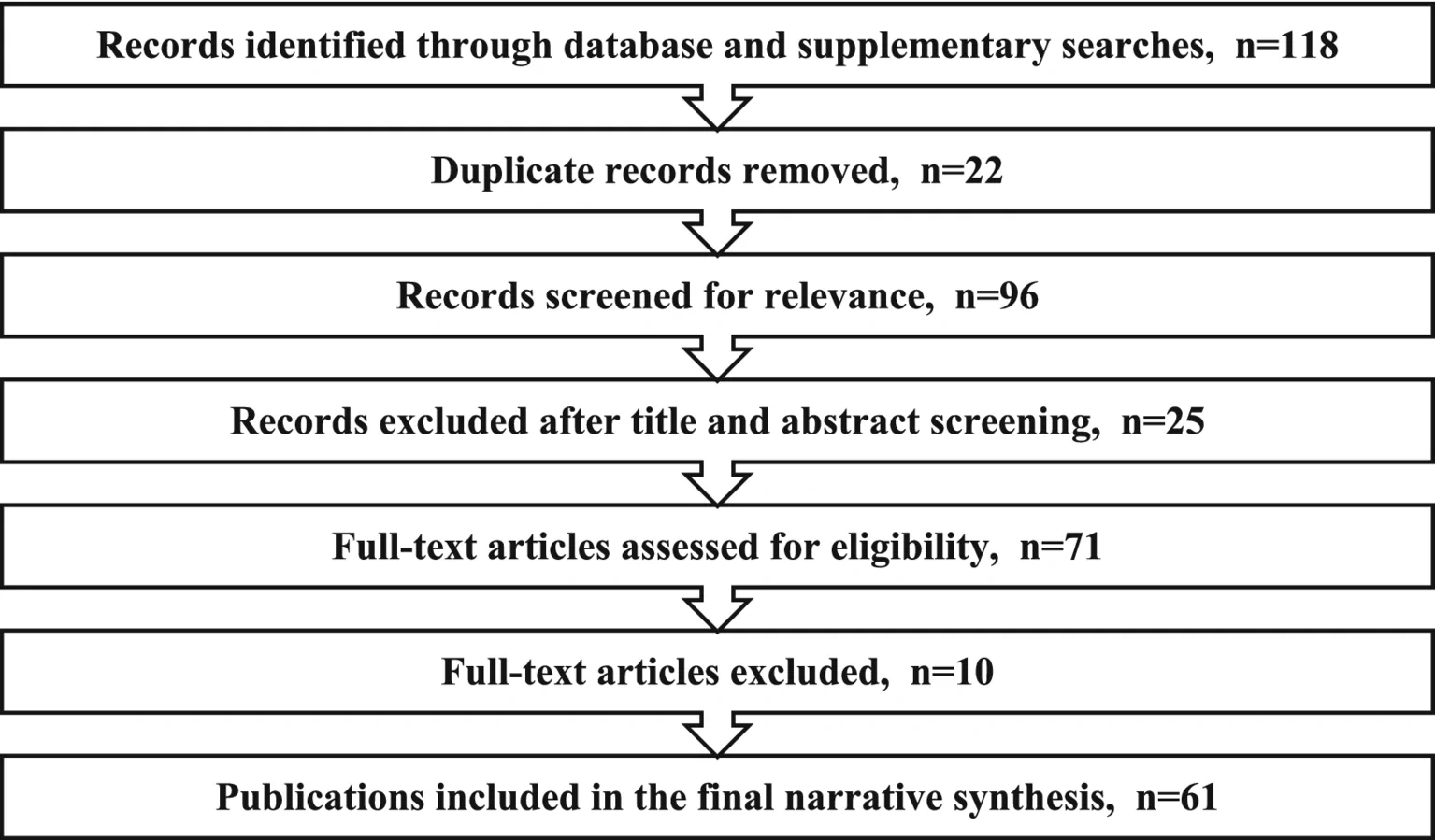
Flow diagram of the literature search and study-selection process.

Owing to differences in study design, exposure assessment, and outcome measures, findings are presented as a qualitative narrative synthesis rather than a quantitative analysis.

## 3 Biological basis of reproductive toxicity

Normal male reproductive function depends on the integrity of the hypothalamic–pituitary-gonadal (HPG) axis, adequate testosterone production, normal spermatogenesis, and preservation of sperm structure and function. The hypothalamic–pituitary–gonadal axis coordinates testosterone production and sperm development through a complex interaction between the hypothalamus, pituitary gland, Leydig cells, and Sertoli cells. Disruption of any component of this system may adversely affect reproductive capacity. Experimental studies have shown that pyrethroid insecticides may interfere with several biological processes involved in male reproduction. One of the most consistently reported findings is the induction of oxidative stress following exposure to pyrethroid compounds (5, 8–10, 20–27). Increased production of reactive oxygen species can overwhelm antioxidant defenses, resulting in lipid peroxidation, cellular injury, and impaired testicular function. Because spermatozoa possess limited antioxidant protection and high concentrations of polyunsaturated fatty acids within their membranes, they are particularly susceptible to oxidative damage.

Pyrethroids have also been reported to exhibit endocrine-disrupting properties. Experimental evidence suggests that some pyrethroid compounds and their metabolites may interact with hormone receptors and interfere with normal endocrine signaling (5, 11, 12). Human studies have further reported associations between pyrethroid metabolites and alterations in reproductive hormone concentrations, including testosterone and other sex steroid hormones (18, 28–36). Such disturbances may adversely affect spermatogenesis and overall reproductive function.

In addition to hormonal effects, pyrethroid exposure has been associated with genetic damage in sperm cells. DNA integrity is essential for normal fertilization and embryo development. Environmental exposure to pyrethroids has been linked to increased sperm DNA fragmentation in human studies, suggesting a potential pathway through which these compounds may impair fertility (19, 37–39).

Collectively, current evidence suggests that lambda-cyhalothrin-induced male reproductive dysfunction is likely multifactorial, involving central and peripheral neurotoxicity, endocrine disruption, oxidative stress, impaired Leydig cell function, sperm DNA damage, and altered spermatogenesis (6–12, 14–17, 40–44). The principal neuroendocrine pathways through which lambda-cyhalothrin may impair male reproductive function, together with the current level of evidence and remaining knowledge gaps, are summarized in Figure 2 and Table 1.

**Figure 2:**
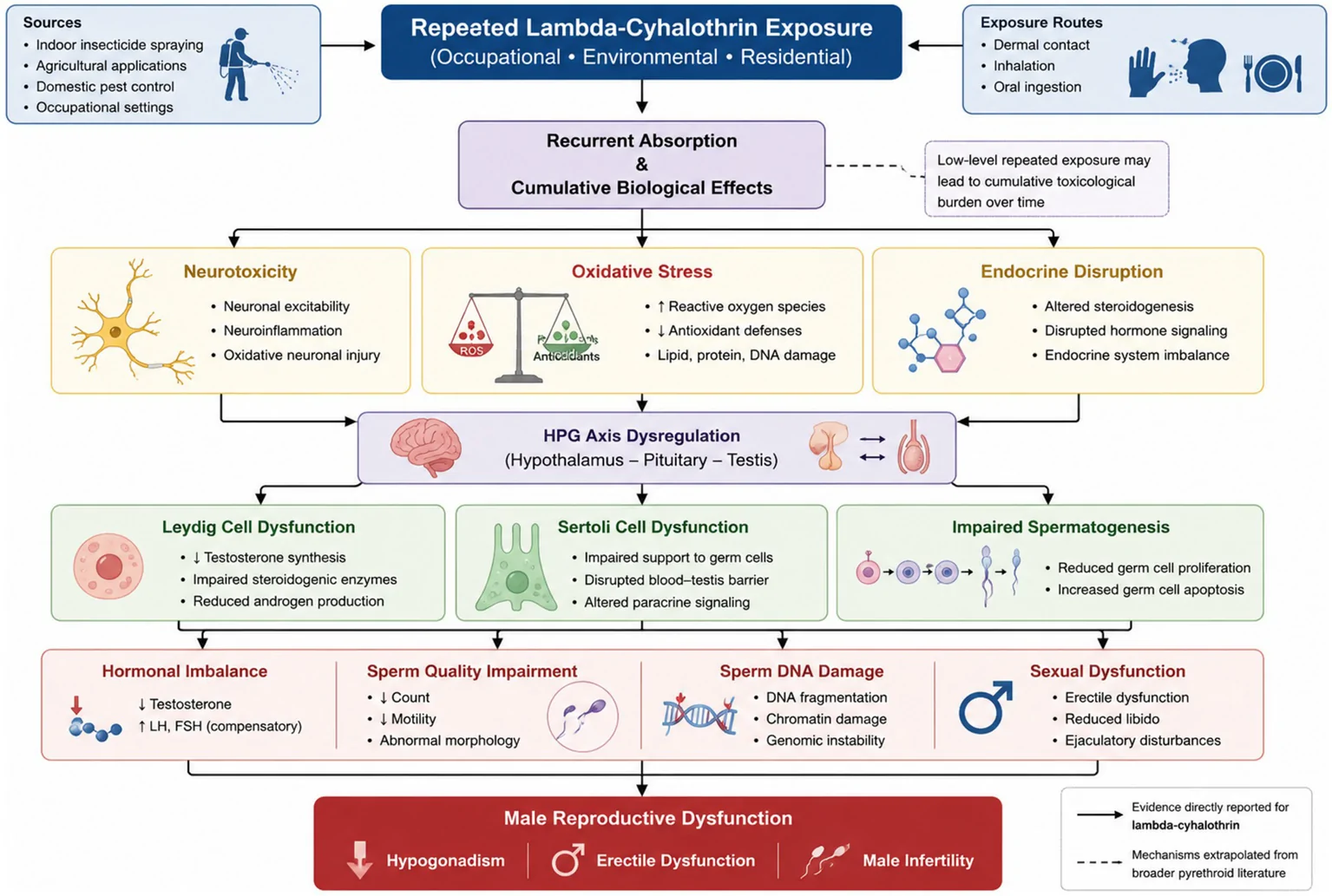
Proposed mechanistic framework linking repeated lambda-cyhalothrin exposure to male reproductive dysfunction. Repeated exposure may lead to recurrent absorption, cumulative biological effects, and progressive impairment of male reproductive function.

**Table 1:** Integrated neuroendocrine mechanisms linking lambda-cyhalothrin exposure to male reproductive dysfunction and current evidence.

Biological pathway	Proposed mechanism	Current evidence	Knowledge gap	Key supporting references
Central neurotoxicity	Altered hypothalamic neurotransmission and hypothalamic–pituitary-gonadal (HPG) axis regulation	Experimental	Direct human neuroendocrine studies	(6, 16, 41)
Autonomic dysfunction	Disturbed autonomic regulation of penile erection and sexual function	Indirect	Human studies evaluating autonomic dysfunction after lambda-cyhalothrin exposure	(6, 42–44)
Peripheral neurotoxicity	Peripheral nerve injury potentially contributing to sexual dysfunction	Clinical neurophysiological confirmation	Experimental	(14, 16)
Endocrine disruption	Altered hormone receptor signaling and impaired reproductive hormone regulation	Experimental + Epidemiological	Longitudinal endocrine studies	(11, 12, 15, 18, 27–36, 40, 45, 47, 54, 58)
Oxidative stress	Excess reactive oxygen species, lipid peroxidation, mitochondrial dysfunction, and antioxidant depletion	Strong	Validation of biomarkers in exposed workers	(5, 7–10, 20–27)
Leydig cell dysfunction	Impaired Leydig cell development and reduced testosterone synthesis	Experimental	Human confirmation	(7, 15, 20, 21, 24, 27, 30, 32–35)
Blood-testis barrier disruption	Increased blood-testis barrier permeability and germ-cell injury	Experimental	Human evidence unavailable	(7)
Impaired spermatogenesis	Reduced sperm production and maturation secondary to hormonal and cellular injury	Experimental	Dose–response relationships in humans	(7, 15, 17, 20–27, 32–35, 48, 49, 51, 53–55, 58, 59)
Sperm DNA damage	Oxidative DNA fragmentation and genomic instability	Experimental + Human	Prospective fertility outcome studies	(7, 19, 27, 37–39, 46, 50, 53, 54, 56, 58)
Hypogonadism	Reduced testosterone resulting from HPG axis and Leydig cell dysfunction	Moderate	Prospective clinical studies	(7, 15, 18, 28–36, 45, 58)
Erectile dysfunction	Combined neuroendocrine and autonomic impairment affecting erectile physiology	Limited	Direct epidemiological evidence	(6, 14, 36, 42–44, 60)
Male infertility	Final consequence of interacting neurotoxic, endocrine, oxidative, and testicular mechanisms	Moderate	Large prospective occupational cohorts	(7, 17, 19–29, 31–39, 45, 46, 48–55, 58)

### 3.1 Molecular mechanisms underlying lambda-cyhalothrin-induced male reproductive toxicity

Current evidence indicates that lambda-cyhalothrin-induced reproductive toxicity is mediated through multiple interconnected molecular pathways rather than a single toxicological mechanism.

Oxidative stress also disrupts intracellular signaling pathways involved in cell survival and inflammation. Increased ROS activates nuclear factor-kappa B (NF-κB) and mitogen-activated protein kinase (MAPK) signaling, promoting the expression of pro-inflammatory cytokines while suppressing endogenous antioxidant responses mediated by the Nrf2 pathway (8–10). Persistent oxidative injury further impairs mitochondrial membrane integrity, leading to cytochrome c release and activation of the intrinsic apoptotic pathway through increased Bax/Bcl-2 ratio and caspase-3 activation, ultimately contributing to germ-cell apoptosis and impaired spermatogenesis (7–10).

Steroidogenesis is another important molecular target. Experimental studies have demonstrated impaired Leydig-cell maturation and reduced testosterone production following lambda-cyhalothrin exposure (7, 15). These alterations are thought to involve disruption of steroidogenic enzymes responsible for testosterone biosynthesis, including steroidogenic acute regulatory protein (StAR), CYP11A1, 3β-hydroxysteroid dehydrogenase (3β-HSD), and 17β-hydroxysteroid dehydrogenase (17β-HSD), resulting in reduced androgen synthesis and secondary hypothalamic–pituitary-gonadal axis dysfunction (11, 12, 15).

In addition to endocrine disruption, recent evidence suggests that lambda-cyhalothrin may compromise the integrity of the blood-testis barrier, a highly specialized Sertoli-cell junctional complex essential for maintaining normal spermatogenesis. Experimental studies have reported structural disruption of this barrier associated with oxidative stress and inflammatory injury, thereby increasing germ-cell vulnerability and impairing the testicular microenvironment (7).

Collectively, these molecular events support a mechanistic cascade in which oxidative stress, mitochondrial dysfunction, inflammatory signaling, apoptosis, steroidogenic impairment, and blood-testis barrier disruption interact to produce progressive deterioration of male reproductive function. Although most mechanistic evidence currently derives from experimental animal models, these pathways provide strong biological plausibility for the hormonal alterations, sperm abnormalities, and reproductive dysfunction observed in human epidemiological studies (7–12, 18, 19, 45, 46).

## 4 Evidence from human studies

Human studies investigating the reproductive effects of pyrethroid exposure have primarily relied on biomonitoring of urinary metabolites, reproductive hormone assessment, semen analysis, and evaluation of sperm DNA integrity. Although the available evidence remains limited, several studies have reported associations between pyrethroid exposure and adverse reproductive outcomes.

Importantly, commonly measured urinary biomarkers such as 3-phenoxybenzoic acid (3-PBA) reflect exposure to multiple pyrethroids and are not specific to lambda-cyhalothrin. Therefore, while human biomonitoring studies support an association between pyrethroid exposure and adverse male reproductive outcomes, lambda-cyhalothrin-specific attribution in humans remains limited.

### 4.1 Reproductive hormones

Alterations in reproductive hormone concentrations represent one of the earliest indicators of impaired male reproductive function. In a study of non-occupationally exposed adult males, Han et al. reported significant associations between urinary concentrations of the pyrethroid metabolite 3-phenoxybenzoic acid (3-PBA) and changes in reproductive hormone levels, suggesting possible disruption of endocrine regulation (18). Similar findings were reported by Meeker et al., who observed associations between pyrethroid metabolites and serum reproductive hormones in adult men (45).

These observations are consistent with experimental evidence demonstrating endocrine-disrupting and antiandrogenic properties of several pyrethroid compounds (11, 12, 47). Nevertheless, the cross-sectional design of most human studies limits causal interpretation.

Experimental studies on lambda-cyhalothrin further support these observations by demonstrating impaired Leydig cell development and disruption of testosterone production, providing biological plausibility for the hormonal alterations reported in human studies (7, 15).

### 4.2 Semen quality

Semen quality remains a cornerstone of male fertility assessment. Several human studies have examined the relationship between pyrethroid exposure and semen parameters, including sperm concentration, motility, and morphology. Meeker et al. reported associations between urinary pyrethroid metabolites and reduced semen quality among men attending an infertility clinic (37, 46, 48–51).

More recently, a systematic review and meta-analysis concluded that pyrethroid exposure was associated with adverse effects on semen quality, although considerable heterogeneity was observed among studies (52–54). Differences in exposure assessment methods, study populations, and outcome definitions may partly explain the variability in reported findings.

Experimental evidence also suggests that lambda-cyhalothrin may reduce reproductive competence through combined effects on spermatogenesis, endocrine regulation, and sperm function (7, 15, 17, 20–27, 32–35, 55).

### 4.3 Sperm DNA integrity

Beyond conventional semen parameters, sperm DNA integrity has emerged as an important marker of male reproductive potential. Jurewicz et al. found that environmental exposure to pyrethroids was associated with increased DNA damage in human sperm cells (19). Similar findings were reported among occupationally exposed workers, where pesticide exposure was associated with higher levels of sperm DNA fragmentation (56).

Because sperm DNA damage may impair fertilization, embryo development, and pregnancy outcomes, these findings provide additional support for a potential relationship between pyrethroid exposure and reduced male fertility. Oxidative stress induced by lambda-cyhalothrin may contribute to this process by increasing reactive oxygen species and compromising genomic stability in sperm cells (7, 8, 10).

### 4.4 Occupational exposure

Occupational exposure generally results in higher and more sustained pyrethroid contact than environmental exposure. Biomonitoring studies have demonstrated measurable pyrethroid metabolite levels among pest-control workers and other occupationally exposed populations (1). Additional studies have confirmed exposure in agricultural and rural settings through both occupational and residential pathways (2). It should be noted that fenvalerate is a non-chrysanthemate type II pyrethroid with toxicokinetic and metabolic properties distinct from lambda-cyhalothrin; therefore, findings from this occupational study (56) cannot be directly extrapolated to lambda-cyhalothrin without acknowledging these structural and toxicological differences.

Occupational exposure represents one of the most relevant scenarios for chronic lambda-cyhalothrin exposure, given the higher and more prolonged contact typically involved.

Most human studies are cross-sectional, rely on urinary metabolites reflecting only recent exposure, include heterogeneous pyrethroid mixtures rather than compound-specific exposure, and cannot establish temporal relationships. Consequently, the apparent association between pyrethroid exposure and male infertility should be interpreted cautiously despite consistent experimental evidence.

The available epidemiological and occupational evidence evaluating the relationship between pyrethroid exposure and male reproductive outcomes is summarized in Table 2.

**Table 2:** Critical appraisal of human evidence investigating pyrethroid exposure and male reproductive outcomes.

Study	Study design	Exposure assessment	Main findings	Key limitations
Han et al. (18)	Cross-sectional	Urinary 3-PBA	Altered reproductive hormone levels	Cross-sectional design; cannot establish causality
Meeker et al. (46)	Cross-sectional	Urinary pyrethroid metabolites	Reduced semen quality	Short-term biomonitoring; mixed pyrethroid exposure
Meeker et al. (45)	Cross-sectional	Urinary pyrethroid metabolites	Associations with reproductive hormone concentrations	Exposure not specific to lambda-cyhalothrin
Jurewicz et al. (19)	Cross-sectional	Environmental pyrethroid exposure	Increased sperm DNA damage	Compound-specific exposure not identified
Bian et al. (56)	Occupational cross-sectional	Fenvalerate exposure	Increased sperm DNA fragmentation	Different pyrethroid; findings cannot be directly extrapolated to lambda-cyhalothrin
Wang et al. (1)	Biomonitoring study	Occupational exposure	Documented occupational pyrethroid exposure	No reproductive outcomes evaluated
Bravo et al. (2)	Exposure assessment study	Occupational and residential exposure	Confirmed multiple exposure pathways	No reproductive outcomes evaluated
Akhigbe et al. (52)	Systematic review & meta-analysis	Multiple human studies	Evidence of adverse associations with semen quality, with substantial heterogeneity among studies	High heterogeneity among included studies
Radwan et al. (28)	Cross-sectional	Urinary pyrethroid metabolites	Associations with semen quality and reproductive hormone alterations	Cross-sectional design; exposure not specific to lambda-cyhalothrin
Xia et al. (48)	Cross-sectional	Urinary pyrethroid metabolites	Associations between pyrethroid metabolites and impaired semen parameters	Single-spot urinary biomarkers; mixed pyrethroid exposure
Ji et al. (37)	Cross-sectional	Urinary pyrethroid metabolites	Reduced semen quality and increased sperm DNA damage	Non-occupational exposure; compound-specific exposure not identified
Yoshinaga et al. (29)	Cross-sectional	Urinary 3-PBA	No consistent association with reproductive hormone levels	3-PBA is non-specific for individual pyrethroids; young healthy population
Imai et al. (49)	Cross-sectional	Urinary 3-PBA	Associations between pyrethroid exposure and selected semen parameters	Cross-sectional design; 3-PBA does not identify the specific pyrethroid
Young et al. (38)	Cross-sectional	Urinary pyrethroid metabolites	Associations with sperm sex-chromosome disomy	Small cross-sectional sample; mixed pyrethroid exposure
Radwan et al. (39)	Cross-sectional	Urinary pyrethroid metabolites	Associations with sperm aneuploidy	Cross-sectional design; compound-specific exposure uncertain
Kamijima et al. (59)	Occupational cross-sectional	Occupational insecticide spraying	Differences in semen indices among insecticide sprayers	Mixed organophosphate and pyrethroid exposure; cannot isolate lambda-cyhalothrin
Meeker et al. (31)	Cross-sectional	Urinary nonpersistent insecticide metabolites	Associations with male reproductive hormone concentrations	Mixed insecticide exposure; cross-sectional design
Perry et al. (50)	Cross-sectional	Urinary pyrethroid and organophosphate metabolites	Associations with sperm concentration	Mixed pesticide exposure; single biomarker measurements
Hu et al. (51)	Cross-sectional	Urinary pyrethroid metabolites	Associations with semen-quality parameters	Cross-sectional design; metabolite-based exposure assessment
Giulioni et al. (53)	Systematic review and meta-analysis	Multiple human studies	Pyrethroid exposure was associated mainly with abnormal sperm morphology; overall semen findings were inconsistent	Few eligible pyrethroid studies and substantial heterogeneity

Overall, the available human evidence is suggestive but remains limited by predominantly cross-sectional study designs, heterogeneous exposure assessment methods, mixed pyrethroid exposure, and limited compound-specific data for lambda-cyhalothrin.

## 5 Discussion

The available evidence indicates a biologically plausible association between chronic lambda-cyhalothrin exposure and male reproductive dysfunction. Experimental studies consistently demonstrate that lambda-cyhalothrin may induce neurotoxicity, endocrine disruption, impaired Leydig cell function, oxidative stress, and testicular injury, ultimately affecting spermatogenesis and male reproductive function (6–12, 14–17, 20, 21, 24, 41–44). Neurotoxic and autonomic effects of lambda-cyhalothrin may plausibly contribute to neurovascular dysfunction relevant to erectile function; however, this proposed pathway has not yet been directly demonstrated in human lambda-cyhalothrin exposure studies. Human studies have also reported associations between pyrethroid metabolites and alterations in reproductive hormone concentrations, semen quality, and sperm DNA integrity (18, 19, 45, 46, 52–54, 56, 61).

Among the reported findings, neurotoxicity, hormonal alterations, sperm DNA damage, and impaired reproductive function appear to be the most consistently observed outcomes. These observations are biologically plausible given the documented ability of pyrethroids to induce oxidative stress and interfere with endocrine signaling (5, 7–12, 25, 27, 35). Such effects may compromise spermatogenesis and sperm function, thereby reducing male reproductive potential. The available evidence also suggests that neurotoxicity and endocrine dysfunction may represent interconnected mechanisms rather than independent toxicological effects, although direct human evidence remains limited (6, 7, 14–17, 41–44).

However, the current evidence remains insufficient to establish a causal relationship between pyrethroid exposure and male infertility. Most human studies are cross-sectional and rely on urinary metabolites that primarily reflect recent exposure. Furthermore, infertility is a multifactorial condition influenced by age, obesity, smoking, alcohol consumption, medical comorbidities, genetic factors, and exposure to other environmental contaminants (13, 57). These factors may confound observed associations and complicate interpretation of epidemiological findings.

Another limitation is the variability in exposure assessment and outcome measurement across studies. Differences in individual pyrethroid compounds, particularly their toxicokinetic and toxicodynamic characteristics, exposure levels, study populations, and laboratory methodologies make direct comparisons difficult and may contribute to inconsistent findings. Moreover, studies directly investigating the relationship between chronic occupational lambda-cyhalothrin exposure, neurotoxicity, hypogonadism, erectile dysfunction, and male infertility remain scarce, representing an important gap in the current literature (1, 2, 5–7, 14–16, 56).

Nevertheless, the widespread use of pyrethroid insecticides and the consistent biological signals reported across experimental and human studies justify continued investigation. Future research should prioritize prospective occupational cohorts evaluating repeated biomonitoring together with neuroendocrine function, reproductive hormones, semen quality, erectile function, and clinically relevant fertility outcomes.

Based on the currently available evidence, chronic lambda-cyhalothrin exposure should be considered a potential contributor to male reproductive dysfunction, although definitive conclusions regarding causality cannot yet be drawn.

### 5.1 Research gap, mechanistic perspective, and novel contribution

This review proposes an integrated mechanistic perspective of lambda-cyhalothrin toxicity by linking evidence across the central, autonomic, and peripheral nervous systems with endocrine dysfunction, hypogonadism, erectile dysfunction, and male infertility. This integrated neuroendocrine framework highlights an important knowledge gap and may provide a foundation for future experimental, occupational, and epidemiological research.

### 5.2 Public health implications

Current evidence supports greater consideration of male reproductive and neuroendocrine health in lambda-cyhalothrin safety assessments. Reproductive and neurotoxic endpoints should be incorporated into pesticide risk evaluation, while occupational exposure monitoring and worker protection measures should be strengthened. Well-designed prospective occupational studies are needed to clarify the long-term risks of chronic lambda-cyhalothrin exposure to male reproductive health (3).

## 6 Critical appraisal of current evidence

The main limitation of the current evidence is the scarcity of prospective human studies specifically addressing lambda-cyhalothrin exposure. Most epidemiological data relate to pyrethroids as a group, making compound-specific interpretation difficult. Although experimental studies provide substantial mechanistic evidence, extrapolation to humans requires caution because of differences in dose, exposure duration, and species. Prospective studies with compound-specific exposure assessment are needed to better define the reproductive effects of chronic lambda-cyhalothrin exposure.

## 7 Conclusion

Current evidence suggests that lambda-cyhalothrin may adversely affect male reproductive health through interconnected neurotoxic, oxidative, endocrine, and testicular mechanisms. Experimental evidence consistently supports these pathways, whereas human evidence remains limited and does not yet establish causality. Well-designed prospective studies with compound-specific exposure assessment are needed to clarify dose–response relationships and determine the reproductive effects of chronic lambda-cyhalothrin exposure.
